# Total knee arthroplasties performed with a mini-incision or a standard incision. Similar results at six months follow-up

**DOI:** 10.1186/1471-2474-11-27

**Published:** 2010-02-06

**Authors:** Daniel Hernandez-Vaquero, Alfonso Noriega-Fernandez, Abelardo Suarez-Vazquez

**Affiliations:** 1 School of Medicine, University of Oviedo, Oviedo, Asturias, Spain; 2 Hospital St Agustin, Aviles, Asturias, Spain

## Abstract

**Background:**

Minimal invasion surgery (MIS) is a recent technique recommended for Total knee arthroplasty (TKA) but demands an effort of the surgeons and the learning curve may be long.

**Methods:**

Twenty six MIS-TKA were matched to 36 standard TKA with respect to age, sex, body mass index or preoperative score. All patients suffered from knee osteoarthritis, which had not improved with medical treatment and which presented a less than 10° deformity in the coronal and sagittal radiographic projections. At six months after the surgery a specific questionnaire was completed as well as the KSS (Knee Society rating scale), the generic short-form health questionnaire (SF-12) and a visual analogue scale (VAS).

**Results:**

The MIS technique required more time of surgery (p < 0.001), hospital stay was noticeably shorter (p < 0.05) and drainage volume collected after surgery was significantly higher in the standard technique. We observe a higher frequency in small sizes implants for MIS surgery but no statistically significant differences were found between both groups regarding the radiological alignment of the implant. At six months no differences were found between the groups in range of motion, KSS scores, the physical or mental subscale SF-12, patient's pain perception, satisfaction or subjective improvement.

**Conclusions:**

Minimal invasion surgery in total knee arthroplasty showed no improvement over a standard approach.

## Background

Total knee arthroplasty (TKA) is a very successful procedure in the treatment of end-stage arthritis or deformity of the knee. Long-term results for pain relief and functional improvement have been excellent. The procedure, however, traditionally requires an extensile approach. The medial parapatellar arthrotomy is the most common method used to expose the knee. This exposure involves patella eversion and generally is done through large incisions of approximately 20 to 30 cm. Although the long-term results of knee arthroplasty have proven to be excellent, the rehabilitation period often is long and painful [1]. In order to improve the patient's well-being in the immediate postoperative period and to lessen the aesthetic impact, other, less traumatic exposures (MIS-TKA) have been introduced, including the subvastus, midvastus, and lateral arthrotomy. The mini midvastus approach extends from the tibial tubercle to the superior patella and then to the muscle of the vastus medialis, and the muscle fibers are not cut [2]. Although recommendations for the use of this technique are not precisely defined yet, the exclusion criteria for patients to receive the MIS-TKA were rheumatoid arthritis, obesity, severe osteoporosis, valgus-varus deformity greater than 10°, previous arthrotomy on the knee and preoperative knee flexion less than 100°. These techniques aim for a faster recovery of mobility, a shorter postoperative period, a reduction in blood loss, less pain throughout the postoperative period, a lessened aesthetic impact, and to reduce the amount of health resources required by resorting to a smaller incision and a less aggressive technique for soft tissues; they will not, however, impair the good results this procedure achieves.

The purpose of this study is to evaluate the short-term functional and health-related quality of life results of MIS-TKA (mini-mid vastus approach) compared with a traditional TKA using a medial parapatellar exposure. Additionally, this study examines the effect of MIS-TKA on operative time, postoperative well-being, health resources used, radiographic alignment and complications.

## Methods

**Figure 1:**
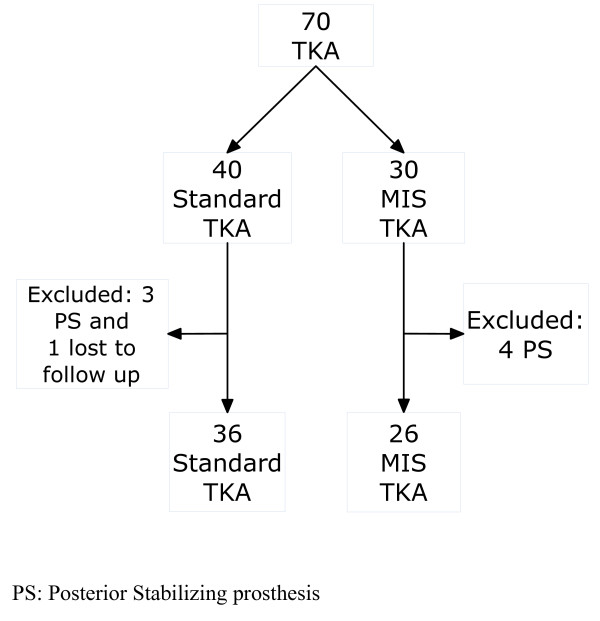
**Flow chart of patients involved in the study**.

This is a prospective and randomized study. Data were collected before surgery, at immediate follow-up and at six months of follow-up. It was calculated that 70 patients would be required in order to determine whether there was a 15 points difference between the two groups in the Knee Society clinical rating system (KSS) [3] at six months. The study population was divided into two groups of patients who underwent surgery with the same surgeons in 2005: 26 MIS-TKA and 36 TKA (Figure 1), following the same arthroplasty model and similar pre and postoperative procedures. Using a table of random numbers, patients would be allocated to either the minimally invasive group (MIS-TKA) (group A) or the standard group (group B) prior to surgery. We explained to the patient the type of surgery in the moment of random assignment. Written informed consent was obtained from all patients, and the study was approved by the regional Ethics Committee. Demographic, clinical, and radiographic data were collected and measured by the authors or their research assistants. All patients suffered from knee osteoarthritis, which had not improved with medical treatment and which presented a less than 10° deformity in the coronal and sagittal radiographic projections. Patients whom already had gone through previous knee surgery other than arthroscopy were excluded.

Preoperative data were similar in both groups (Table 1). No statistically significant differences were found for the body mass index (BMI) (p: 0.32), age (p: 0.86), gender (p: 0.94), previous arthroscopy frequency or preoperative score according to the KSS (p: 0.78). The MIS-TKA technique was introduced in our hospital back in 2004. 17 interventions were undertaken, so it was determined that the learning curve had been properly completed, and that there was enough experience so as to compare the results for both techniques. All knee prosthesis were Triathlon™ cruciate retaining (CR) (Stryker Orthopaedics, Mahwah, NJ, U.S.A.) as primary surgery. The Triathlon™ Total Knee System is made of cobalt chrome with modular tibial and femoral inserts; both cemented and noncemented (periapatite-coated) versions are available. All the implants used in this study were cemented, and all patients had patella resurfacing. A first-generation cephalosphorins antibiotic prophylaxis was used during preoperative care, and low-molecular-weight subcutaneous heparins were administered as antithrombotic treatment for six weeks after surgery.

**Table 1:** Preoperative Data

	MIS-TKA	Standard
Number of TKAs	26	36

Women/men	21/5	30/6

Age (years)	70.8 (SD, 5.9)	70,5 (SD, 6.9)

BMI (mean)	32.1 (SD, 6)	30.8 (SD, 3.3)

Previous surgery (*)	15%	16%

Extension (degrees)	4 (range, 0--12)	6 (range, 0--20)

Flexion (degrees)	100 (range, 85--130)	106 (range, 75--132)

KSS (points)	81	79

(*) Arthroscopy

**Figure 2:**
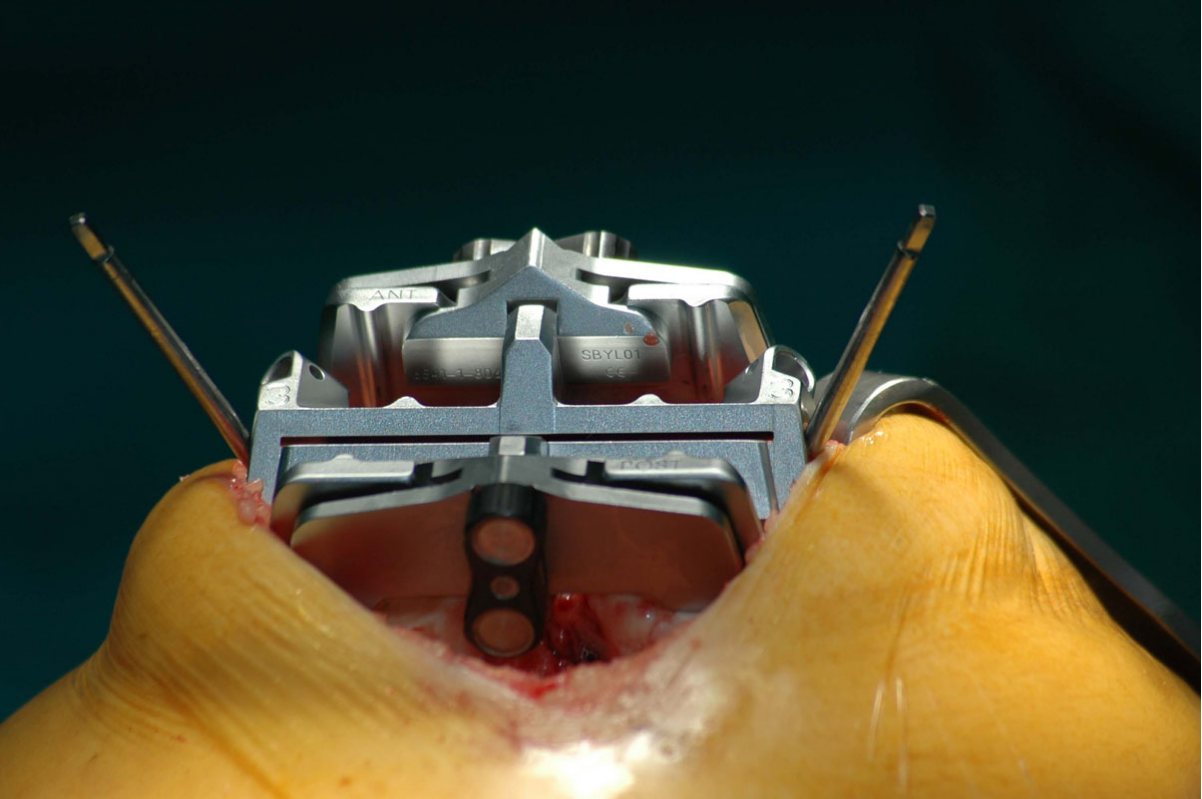
**4-in-1 cutting block**.

**Figure 3:**
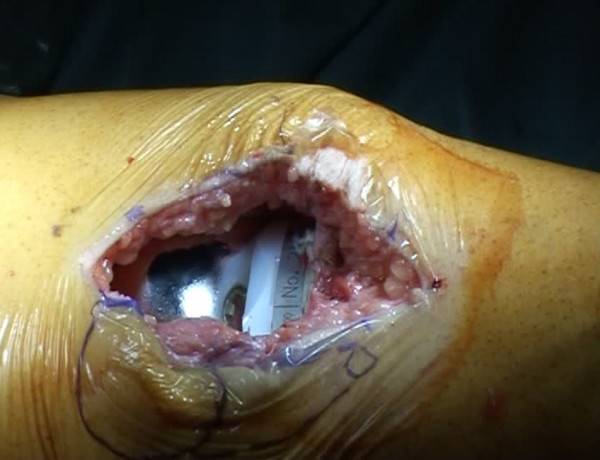
**Arthroplasty performed by MIS**.

The skin incision in the mini mid-vastus Group was made along the medial aspect of the patella and from 0.5-1 cm proximal to the superior pole of the patella to approximately 2-4 cm beyond the medial extent of the tibial tubercle. The vastus medialis obliquus muscle was split approximately 2 cm in line with its fibers from the superomedial pole of the patella, but rip frequently appears increasing the size of vastus incision (in three patients the final length was 3 cm and 3.5 cm in other two). Distally, the medial parapatellar retinaculum was incised and the incision was continued medial to the patellar tendon to the tibia approximately 5 mm medial to the tubercle. The knee was flexed and the patella was subluxed, but not everted, and the technique "mobile windows" was carried out so as to better visualize the femorotibial compartments. The femoral and tibial incisions are performed with the specific equipment (Figure 2). In the standard group, intramedullary guides were used for the distal femoral cut, with a target alignment of 5° to 6° of valgus with 0° to 3° of flexion. The proximal tibia was cut using extramedullary guides at right angles in the coronal plane and at 3° of posterior slope. After these cuts were made, all remaining posterior osteophytes and meniscal remnants were removed. Ligament balancing was accomplished using either spacer blocks or a tension/balancer. Final preparation was performed with a 4-in-1 cutting block. The wound was closed routinely (Figure 3) with 1 drain in the group A and 2 in the group B. A tourniquet was used in all cases and deflated routinely before the wound closes.

A specific questionnaire was filled in, in which 14 variables were compiled (Table 2) and the KSS scale was filled in at six months after the intervention, along with the generic short-form health questionnaire (SF-12) and a visual analogue scale (VAS), rated between 0 and 10, to measure the satisfaction, pain and subjective improvement. The SF-12 and VAS questionnaires were completed by the patients without assistance from the physician or office staff to ensure that all responses were based entirely on self assessment. Data were processed with Microsoft Excel 2002. A univariate and vibariate analysis was carried out with the Statistical software application EPI 6, using the anova, chi-square and t-Student tests.

**Table 2:** Data collected

Surgery duration (min)
Size of incision (cm)

Size of the femoral component

Size of the tibial component

Thickness of the tibial polyethylene insert (mm)

Length of post-operative hospital stay (days)

Blood loss in the immediate post-operative check-up (grams of hemoglobin)

Redon draining (cc)

Number of rescue analgesic doses during immediate post-operative period

Radiographic alignment of the tibial component of the prosthesis/axis on the tibia (degrees)


Radiographic alignment of the femoral component of the prosthesis/axis on the femur (degrees)

Radiographic measurement of the femorotibial axis (degrees)

Use of walking stick or crutches (one, two, none)

Complications

## Results

### Surgery

Mean time of the surgery for Group B was 103.19 minutes (SD 20.94), while the mean time for Group A was 130 minutes (SD 26.94). This means applying the MIS technique required 26% more time (p < 0.001). The mean size of the length of the cutaneous incision was 11 cm (8-12) for the MIS-TKA Group, and 19 cm (17.5-23) for the Group using the traditional method.

**Figure 4:**
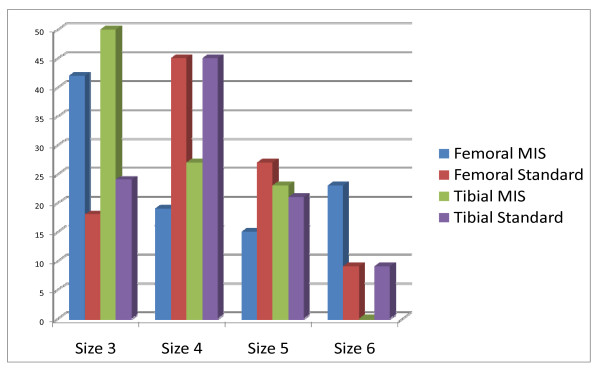
**Size of implants (%)**.

At the time of the experiment (2005), we only had access to sizes 2,3,4,5, and 6 of the implanted model. The sizes of the most frequently used components were sizes 4 and 5, although in the MIS-TKA Group they were sizes 3 and 4 (Figure 4). We observe a higher frequency in size 3 implants for MIS surgery than for traditional surgery, both for the femoral (p < 0.03) and for the tibial (p < 0.05). No significant differences were found in the width of the polyethylene insert for both Groups (36% 9 mm, 45% 11 mm and 18% 13 mm for the standard technique and 53% 9 mm, 39% 11 mm and 7% 13 mm for the MIS Group).

### Immediate post-operative period

Hospital stay was noticeably shorter (p < 0.05) for the MIS Group patients (mean: 6.92; SD 1.47) than for the standard Group (mean: 7.88; SD 2.06). The reduction of hemoglobin grams lost in the immediate post-operative period for the MIS Group was 1.87 (mean; SD 0.80), whereas in the other Group the average loss was 2.95 grams (mean; SD 0.80), which is significantly higher (p < 0.001). Drainage volume collected after surgery for Group A was a mean of 621.15 cc (SD 412.22), whereas the figure for the traditional technique was significantly higher, with a mean of 1072.5 cc (SD 440.64).

The number of paracetamol rescue doses set by our hospital for these kind of surgery (which are necessary for the analgesic control during the two days following surgery) was less for those patients belonging to the MIS-TKA Group (mean: 1; SD 1.23) than for those patients who underwent the conventional technique (mean: 1.83; SD: 1.3), showing a significant difference (p < 0.05).

All patients underwent radiographic post-operative evaluations. No statistically significant differences were found between both groups regarding the alignment of the femoral and tibial component in relation with the femoral and tibial mechanical axis (Table 3). The total series mean tibiofemoral alignment in the coronal plane was 5.2° valgus (range 3° to 8°). Data for the MIS group were: 5.4° valgus (SD: 3.31) and 2.5° flexion (range 0° to 4°) in the sagittal plane, and 4.6° valgus (SD: 2.98) and 2.3° flexion (range, 0° to 2°) in the standard Group, with no significant differences.

**Table 3:** Radiographic alignment of implants

	Femoral component (SD)	Tibial component (SD)	Total tibiofemoral coronal (SD)	Total tibiofemoral sagital (SD)
MIS	+ 5.7° (2.81)	- 1° (2.46)	+ 5.4° (3.31)	2.5° (2.2)

Standard	+ 5.5° (2.68)	- 1.6° (2.08)	+ 4.6° (2.98)	2.3° (2.09)

+: Valgus, -:Varus

### Evaluation at six months

Regarding the observed mobility at six months after surgery, no significant differences were observed between both groups. Extension in the MIS Group was -0.96° (SD 2.46), and -0.97° (SD 2.34) in the standard Group. Mean flexion for the MIS Group was 99.62° (SD: 14.55), whereas it was 99.44° (SD 15.3) in the standard group. There were no severe complications which might have altered the clinical or radiographic results. No progressive radiolucencies were found. Care was taken to position patients as uniformly as possible. However, radiographs were obtained without benefit of fluoroscopy, so the true incidence of radiolucent lines beneath the components cannot be stated with certainty.

The percentage of cutaneous complications, all of them slight and easily cured, was similar between both group (11% in the conventional technique group and 11.5% in the MIS group) but delayed wound healing and local soreness was observed in 6% of patients in the MIS group. 15% of the patients who underwent the MIS technique were using a walking stick at six months of follow-up, whereas in the standard technique group that percentage reached 21%, with no statistically significant differences between groups.

Pain as measured with the VAS scale at six months was 2.2 (SD 1.4) for both Groups. Average satisfaction with the same scale was 8.3 (SD 1.9) for the MIS group and 8.2 (SD 1.3) for the traditional technique Group. No statistically significant differences were found, either. At six months of follow-up, the MIS Group scored a mean of 43.9 (SD 10.5) in the physical subscale of the SF-12, whereas the traditional technique Group scored a mean of 44.9 (SD 97.3), with no significant differences. The same happened with the mental subscales: 45.8 (SD 13.4) for the MIS Group and 50.1 (SD 11.4) for the traditional technique Group. KSS scores were 163.4 (SD 31.4) for the MIS Group ad 162.6 (SD 21.4) for the standard technique Group. This difference was not statistically significant.

## Discussion

There have been many studies which have determined the effectiveness of TKA in reducing pain and deformity and improving functionality. Standard TKA has led to consistent, reproducible, and enduring results. Long term success at 10 years or more encompasses survivorship of greater than 90% for many studies, and more than of 80% of patients were satisfied [4]. For many years, clinicians relied exclusively on objective and physical measures of disability in order to assess orthopedic surgical outcomes. Most have assessed outcomes using standardised knee scoring systems such as KSS, but they have poor internal reliability and small effect sizes, and are therefore not good for assessing outcomes in TKA. It seems necessary to add another kind of evaluation, such as a visual scale and a generic questionnaire on quality of life. Works such as that of Bullens et al [5], show how a comparison between the subjective and objective outcome systems revealed only poor correlations, and this comparison suggests that the concerns and priorities of patients and surgeons can differ.

The outcome measures which we have used (SF-12) is a reliable and validated scoring system which has been used to assess the outcomes of the TKAs [6,7]. It measures generic health concepts so as to allow for the comparison of several groups. It includes eight variables commonly used in health-related research: physical functioning, bodily pain, general health perception, vitality, social functioning, emotional role and mental health. Results are expressed in two subscales, with scores between 0 and 100, designed to have a mean of 50 and a SD of 10 for the general population. VAS provides additional information about subjective outcome after TKA [5] and is used frequently in health. We advocate the use of the patient satisfaction VAS system, in addition to the existing evaluating systems.

Few studies exist which have compared prospectively two groups of patients [8-14], one with MIS, one with the standard technique (Table 4) and, to our knowledge, no study has been published which has prospectively assessed patient-perceived outcomes after MIS-TKA and compared them with standard TKA. Thus, our study offers an original methodology: it compares both groups via functional scales, subjective impressions and opinions on quality of life, which is important to assess a technique proposed to enhance patient's satisfaction and cosmetic benefits. Furthermore, it is a prospective, randomized study in which the same type of arthroplasty was implanted by the same surgeons.

**Table 4:** Some prospective studies MIS-standard TKA

Author, year	Number of TKA
Kelly [8], 2007	21/21

Chin [9], 2007	30/30

Kolisek [10], 2007	40/40

Karachalios [11], 2008	50/50

Kim [12], 2007	120/120

Tahiro [13], 2007	24/25

Han [14], 2008	15/15 (*)

Present serie	26/36

(*) Simultaneous bilateral TKA

The mean operative duration from incision to closure was significantly longer in group MIS-TKA, (26% more); this was already referred to in other studies on MIS in ATR [15-18]. Analysing the size of the implants shows that the MIS group requires smaller components more frequently. Although we haven't found evidence in the literature on this finding, this could be due to the smaller size of the incision or to difficulties when measuring those components. We do not know whether that difference may have an eventual impact. Similarly, the width of the polyethylene tray showed that knees treated with MIS tend to require less wide components, although there appears to be no statistical significance.

Hospital stay for patients in Group A was at least one day less than that of patients who underwent the traditional technique. Other authors do mention this circumstance [19] which helps to make good use of health resources. Blood loss was significantly lower in the MIS Group, both in terms of hemoglobin grams and in the amount of drained blood but the difference in the number of drains in each group could produce a bias in the results of our study. However these data are also in keeping with other studies [8,10] and are supposedly related to the less aggressive surgery. In any case, none of our patients needed to get a blood transfusion.

It has been suggested that minimally-invasive techniques result in less post-operative pain and a reduced requirement for analgesics. In our study, post-operative pain was indeed lower in the MIS Group and, even though it is difficult to measure this variable, we can at least ascertain that this group required fewer analgesics; without patients and nurses blinded to the procedure, there is too much bias to make much of this finding. In other studies on the MIS-TKA technique, analgesic used are measured as en equivalent with morphine [15], according to the total amount of analgesics used [16], or via visual pain scales.

We encountered no major perioperative complications in either group. We observed a greater degree of transitory tumefaction in the edges of the wound in the MIS group; this can be related to the use of separators, and other authors have also mentioned an increase in local wound problems (4 vs. 1) [10]. It is possible that being more careful with the soft parts may had avoided the high number of cutaneous complications.

The alignment of the components measured radiographically with respect to each bone segment to both of them was excellent in both groups. Some authors compare the radiologic outcomes of total knee arthroplasty using the conventional technique with those using MIS techniques. Results were comparable between the mini and control groups [20], but other authors [11] showed that technical errors and higher rates of outliers in postoperative alignment were observed. It is possible that using computer assisted surgery may add some advantages to this technique, bringing a special vision of the bone cuts, thus easing their reproducibility and avoiding outlier cases [21].

At six months of follow-up, results for both groups are similar, and there are no statistically significant differences in none of the parameters. The range of motion in both groups is good at six months; these data are in keeping with other authors, and there is no improvement in recovery in group TKA-MIS, as mentioned in other studies [13,22] where the minimally invasive technique positively contributes to the early restoration of quadriceps strength and a speedy return to normal functioning. In other studies, manipulation was necessary in 14% of the traditional group compared with 2% in the minimal incision group [23] but there was no significant difference in range of motion or functional outcome at 1 year after surgery nor was no significant difference in component position or complication rates.

Variables measured with VAS did not show significant differences between both groups in neither of its two subscales (physical and mental). Both groups reported a good quality of life at six months. On the KSS scale, both groups achieved high scores, but there were no statistically significant differences between them.

Overweight (BMI>40), muscular hypertrophy, previous surgery or low patella are considered factors which may hinder this kind of surgery. In our experience, none of them have posed any problem whatsoever nor had any negative impact on surgery.

It is usual that studies appear showing better short-term outcomes for MIS-TKA [24] regarding the patient's well-being and hospital stay. However, in order to properly gauge the scientific evidence on MIS techniques in ATR, it should be noted that those results have frequently been obtained from expert centers or from surgeons devoted to such techniques [25]. This fact may distort the results, since any complications and the follow-up of TKAs implanted via MIS in general hospitals or non-specialized centers are not analysed. It is possible then that, presently, the MIS technique is more a personal choice of the patient or a commercial demand than a true advancement in TKA placement. The surgeon should look for the minimum size necessary for him to correctly implant a TKA, and look away from showing off he is able to implant arthroplasties with ever smaller incisions. Therefore we recommend the use of a standard arthrotomy with the shortest possible invasion and skin incision. MIS-TKA demands an effort on the part of the surgeon [26], the learning curve may be unacceptably long for a low-volume arthroplasty surgeon [27], it takes more intervention length, require longer tourniquet time and special tools are needed to insert the implant.

## Conclusions

Our study allows us to assert that the MIS technique does offer some advantages for the immediate post-operative period, including a lesser amount of analgesics, and a shorter hospital stay. However, results at six months after surgery are similar, both in functional assessment and in quality of life for the patient.

## Competing interests

Non financial competing interests. This study belongs to a project promoted by the Health Research Institute (Carlos III Health Institute) of the Spanish National Healthcare System.

## Authors' contributions

DHV conceived of the study, and participated in its design and coordination, ANF participated in the design of the study and acquisition of data, ASV participated in analysis and interpretation of data. All authors read and approved the final manuscript.

## Pre-publication history

The pre-publication history for this paper can be accessed here:

http://www.biomedcentral.com/1471-2474/11/27/prepub
